# Safety and effectiveness of vascular closure devices in interventional radiological procedures

**DOI:** 10.1177/15910199221100628

**Published:** 2022-05-11

**Authors:** Esther Kim, Bruno Goncalves Sebastiao, Amy Lee, Sudharshan Ande, Jai Shankar

**Affiliations:** 1 Department of Radiology, Max Rady College of Medicine, Rady Faculty of Health Sciences, 423134 University of Manitoba, Winnipeg, Manitoba, Canada

**Keywords:** Vascular closure device, femoral artery, interventional radiology, complications, femoral access

## Abstract

**Background:**

Although it is well known that vascular closure devices (VCD) are commonly used in therapeutic interventional radiological procedures, standard use in diagnostic procedures is not as well studied.

**Purpose:**

The aim of this study was to determine the real-world safety and effectiveness of the VCD in both diagnostic and therapeutic interventional radiological procedures.

**Materials and methods:**

A retrospective, single center study included all patients where VCDs were used for either a diagnostic or therapeutic interventional procedure. Various demographic and clinical risk factors were recorded and examined for any significant association with successful deployment and complications.

**Results:**

A total of 2072 patients were included. VCDs were successfully deployed in 95.2% of the patients with 4.8% of perioperative complications, which included minor oozing from the puncture site, small hematoma less than or equal to 5 cm, large hematoma greater than 5 cm, pain, and loss of vascular access. Therapeutic (vascular interventional radiology (VIR) and neuro-interventional radiology (NIR)) procedures (OR 3.03, 95% CI 1.51–6.09, *p* = 0.002), use of Angioseal (OR 5.26, 95% CI 3.13–8.33), *p* < 0.001), and no use of antiplatelet medications (OR 0.47, 95% CI 0.22–0.97, *p* = 0.041) were independently associated with successful deployment of VCDs when controlled for other risk factors. Smoking (OR 3.50, 95% CI 2.00–6.05, *p* = <0.001), use of antiplatelet (OR 2.01, 95% CI 1.04–3.87, *p* = 0.037) and use of heparin (OR 1.78, 95% CI 1.10–2.86, *p* = 0.018) were independently associated with higher complication rates.

**Conclusion:**

VCD's were successfully deployed in 95.2% of the patients with 4.8% of perioperative minor complications.

## Introduction

More than 7 million percutaneous procedures are performed worldwide each year.^1^ Recent practice for vascular procedures has shifted towards percutaneous endovascular interventions, most commonly through the femoral artery. Manual compression has been the traditional method for hemostasis of the puncture site. However, it is time consuming, personnel intensive, and requires prolonged bed rest for the patient (up to 4–6 h).^1^ Therefore, there has been increasing popularity in the use of vascular closure devices (VCDs) to achieve hemostasis, as opposed to manual compression. The reasons include faster hemostasis, earlier ambulation, and improved patient comfort, which in turn reduces length of hospital stay and also expedites patient discharge.^2^ Commonly reported minor puncture site complications include bleeding, hematoma, vessel occlusion, and aneurysm.^3^

VCDs are routinely used in therapeutic procedures in both neuro-interventional radiology (NIR) and vascular interventional radiology (VIR). However, at our center, we implemented the use of VCDs in diagnostic NIR and VIR procedures as well, which is a different practice when compared to most other centers.

The purpose of the study was to determine the real-world effectiveness and safety of VCDs in diagnostic and therapeutic interventional procedures.

## Materials and methods

The study was approved by the institutional research ethics board (REB number-HS23663). A retrospective review was conducted to examine deployment success and complication rates in patients undergoing both diagnostic and therapeutic NIR and VIR procedures where VCDs were used to achieve hemostasis at our center. Patients were identified from our interventional radiology database by using the procedure accession numbers where VCDs were used. All consecutive adult patients (18 years or older) where VCDs were used for vascular puncture site closure between January 2016 and December 2019 were included in the study. These included both diagnostic and therapeutic procedures in NIR and VIR. All types of VCDs used were included in our study. Patients under the age of 18, missing data, or with duplicate studies in Picture Archiving and Communication System (PACS) were excluded.

VCDs were initially used primarily for therapeutic NIR cases. A practice change was implemented in our institution just before 2016 and the use of VCDs were extended to all endovascular procedures including diagnostic as well as therapeutic cases. In our institution, the average duration for bed rest after transfemoral procedures is 4–6 h after manual compression and 2 h for those where VCDs are used.

The detailed demographic and clinical data were collected from the progress notes scanned in PACS. The data points included the date of study, operator, type of VCD used, type of procedure, and any immediate or late complication from the VCD. For patient demographics and clinical information, we collected age, binary gender, past medical history (in particular, chronic kidney disease, diabetes mellitus, dyslipidemia, hypertension, obesity, and smoking), anticoagulation/antiplatelet medications, peri-operative use of heparin, lab values (eGFR, INR, platelets, and hemoglobin), and stroke data (tissue plasminogen activator if given and side of clinical deficits, if any). The data was collected by 4 different researchers (EK, BGS, AL, SA), with each collecting data for one calendar year.

The study examined the rate of successful VCD deployment and rate of associated punctures site complications. Failure of the VCD was considered when the device could not be successfully deployed as per information in the device use manual and needed manual compression for 20 min or longer to achieve puncture site hemostasis. The success of deployment was assessed by the operators themselves.

Complications were classified into immediate and delayed complications. Immediate complications were those observed on the table or within 24 h of the procedure and immediate complications were further divided into minor and major. Minor complications did not need any further interventions and included oozing, small hematoma (<5 cm), large hematoma (>5 cm), hematuria, pain, emesis, hives, and bruising. Major complications were those where additional interventions were needed to treat and included loss of access and dissection. These complications were assessed by the operators themselves and documented by the nurse in the progress note.

Delayed complications were defined as those noticed beyond 24 h from the procedure. Patients had variable follow ups depending on their clinical conditions. It was assumed that the patients with delayed complications will present back to the hospital within the first 2 weeks of use of VCDs and will undergo either a focused ultrasound or CT scan or digital subtraction angiography (DSA) of the abdomen and pelvis. Therefore, to assess for delayed complications, we searched the PACS, for any follow up ultrasound or CT angiogram or DSA of the abdomen and pelvis done within 14 days of VCD-use. Patients with delayed complications such as a retroperitoneal hemorrhage, distal thrombus, or aneurysm/pseudoaneurysm were documented.

### Statistics

Descriptive statistics and chi-squared tests were used to compare the different proportions for categorical variables. In patients where VCDs were used bilaterally and VCD failed on at least one side, they were included in the failed category for statistical analysis. Logistic regression was used to calculate the odds ratio for binary variables in univariate analysis. Variables that showed significant association with either deployment of VCD or complications, were included in the multivariate analysis using logistic regression. A *p* < 0.05 was considered significant. Stata 13.0 statistical package was used for statistical analysis.

## Results

From January 2016 to December 2019, a total of 2678 study accession numbers were found to be associated with the use of VCDs in the interventional radiology database. Of these, 596 accession numbers were duplicate accession numbers and were excluded. Ten patients were under the age of 18 and were also excluded from our final list of patients. The final analysis included 2072 patients. Table 1 summarizes the demographic and baseline clinical characteristics of the patients included in our study. The most common chronic diseases were hypertension (42.7%) and diabetes (30.8%).

**Table 1.** Demographic and baseline clinical characteristics of all patients where VCDs were used to achieve hemostasis between January 2016 and December 2019.

Total # of Patients	2072
Age	63 ± 15.9
*Sex*	
Male	1186 (57.2)
Female	886 (42.8)
*Past medical history*	
Chronic kidney disease	123 (5.9)
Diabetes	639 (30.8)
Dyslipidemia	409 (19.7)
Hypertension	884 (42.7)
Obesity	16 (0.8)
Smoking	293 (14.1)
*Blood work*	
eGFR	52.4 ± 16.2
Hemoglobin	125.6 ± 31.3
INR	1.1 ± 0.2
Platelets	254.9 ± 107.6
Anticoagulation	14 (0.7)
Antiplatelet	127 (6.1)
Peri-operative use of heparin	324 (15.6)

Values are presented as number (percentage).

A total of 1302 patients underwent diagnostic procedures, and 743 patients underwent therapeutic procedures (Table 2). The VCDs included in our institution included Angio-Seal VIP (6F and 8F), Angio-Seal Evolution 6F (Terumo Medical Corporation, NJ), Perclose 6F (Abott Vascular, Redwood City, California), and StarClose 6F (Abott Vascular, Redwood City, California). Of these, the 6F Evolution Angio-Seal vascular closure was the most commonly (34%) used device. There was no significant difference in complication rates (*p* = 0.85) and deployment success (*p* = 0.87) between the 6F (n = 1,943 and 8F (n = 227) devices. VCDs were successfully deployed in 95.2% of cases. Patients coming from the emergency room had the highest success rate (99.1%, *p* = 0.023) of VCD deployment.

**Table 2.** Risk factors and their association with complications and successful deployment of vascular closure devices.

	Complications	Odds ratio (95% CI)	*p*-value	Successful Deployment	Odds ratio (95% CI)	*p*-value
*Type of procedure*			0.070			<0.001
Diagnostic NIR (n = 430)	13 (3.0)			407 (93.6)		
Therapeutic NIR (n = 436)	22 (5.1)			426 (96.4)		
Diagnostic VIR (n = 872)	53 (6.1)			812 (92.2)		
Therapeutic VIR (n = 307)	11 (3.6)			287 (91.4)		
*Diagnostic vs Therapeutic*		0.87 (0.57, 1.34)	0.525		2.92 (1.17, 5.13)	<0.001
Diagnostic (n = 1302)	66 (5.1)			1219 (94.2)		
Therapeutic (n = 743)	33 (4.4)			713 (97.9)		
*NIR vs VIR*		1.36 (0.89, 2.08)	0.150		0.57 (0.36, 0.89)	0.001
NIR (n = 866)	35 (4.0)			833 (96.9)		
VIR (n = 1179)	64 (5.4)			1099 (94.6)		
*Type of device*		1.06 (0.90, 1.24)	0.509		0.70 (0.58, 0.84)	<0.001*
Vascular Angio-Seal 6F (n = 604)	25 (4.1)			581 (94.5)		
Vascular Angio-Seal 8F (n = 214)	13 (6.1)			205 (95.3)		
Evolution Angio-Seal 6F (n = 703)	35 (5.0)			675 (94.7)		
Perclose 6F (n = 430)	20 (4.7)			379 (87.1)		
Starclose 6F (n = 94)	6 (6.4)			92 (97.9)		
*Manufacturer of VCD*		1.32 (0.82, 2.11)	0.253		0.29 (0.19, 0.45)	<0.001
Abbott Vascular Inc. (n = 529)	24 (5.0)			471 (89.0)		
Terumo (n = 1543)	58 (3.8)			1461 (94.7)		
*Femoral puncture site*		1.47 (0.95, 2.27)	0.087		0.49 (0.32, 0.76)	<0.001
Left (n = 489)	31 (6.3)			339 (92.8)		
Right (n = 1540)	68 (4.4)			1477 (96.4)		
*Patient location in the hospital*		1.09 (0.76, 1.56)	0.653			0.023
Inpatient (n = 440)	19 (4.3)			416 (95.6)		
Outpatient (n = 1375)	68 (5.0)			1291 (95.0)		
Emergency (n = 223)	11 (5.0)			219 (99.1)		
*Clinical history*						
Chronic kidney disease	7 (5.7)	4.35 (0.92, 20.64)	0.065	112 (91.0)	0.63 (0.15, 2.71)	0.537
Diabetes	32 (5.1)	0.97 (0.63, 1.50)	0.886	590 (92.3)	0.64 (0.41, 0.99)	0.046
Dyslipidemia	21 (5.2)	1.01 (0.61, 1.65)	0.981	381 (93.2)	0.78 (0.47, 1.28)	0.326
Hypertension	38 (4.3)	0.71 (0.46, 1.07)	0.104	826 (93.4)	0.90 (0.58, 1.39)	0.632
Obesity	0 (0.0)	#		15 (93.8)	0.71 (0.09, 5.47)	0.744
Smoking	17 (5.8)	3.50 (2.00, 6.05)	<0.001*	277 (94.5)	1.00 (0.61, 1.66)	0.880
Anticoagulation	1 (7.1)	1.51 (0.20, 11.72)	0.689	13 (92.9)	0.60 (0.08, 4.66)	0.624
Antiplatelet	11 (8.8)	2.01 (1.04, 3.87)	0.037*	115 (90.6)	0.51 (0.26, 1.00)	0.047
Peri-operative use of heparin	24 (7.5)	1.78 (1.10, 2.86)	0.018*	296 (91.4)	0.53 (0.32, 0.86)	0.009

Values are presented as number (percentage).

# Odds ratio could not be calculated as there were zero complications in those who had obesity.

Chi-squared test was used to calculate *p* value.

Odds ratio were calculated using univariate logistic regression analysis.

OR, Odds ration; NIR, Neuro Interventional Radiology; VIR, Vascular Interventional Radiology.

Results of univariate analysis with odds ratio (OR) and their 95% confidence interval (CI) are summarized in Table 2. The binary risk factors (diagnostic/therapeutic, NIR/VIR, VCD manufacturer, puncture side, diabetes, use of antiplatelet medications and heparin) were included in the multivariate analysis to assess their association with successful deployment. Therapeutic (VIR or NIR) procedures (OR- 3.03, 95% CI 1.51; 6.09, *p* = 0.002), Angioseal (Terumo Inc) (OR- 5.26, 95% CI- 3.13; 8.33, *p* < 0.001), and no use of antiplatelet medications (OR- 0.47, 95% CI- 0.22; 0.97, *p* = 0.041) were independently associated with successful deployment of VCDs when controlled for other risk factors. Right puncture side (OR- 1.61, 95% CI- 0.95; 2.70, *p* = 0.074) and no use of heparin (OR- 0.61, 95% CI- 0.35; 1.06, *p* = 0.077) also showed a trend towards significance for successful deployment of VCDs.

The binary risk factors (NIR/VIR, puncture side, smoking, use of antiplatelet medications, and heparin) were included in multivariate analysis to assess their association with complications. This showed that smoking (OR 3.50, 95% CI 2.00–6.05, *p* = <0.001), use of antiplatelet (OR 2.01, 95% CI 1.04–3.87, *p* = 0.037) and use of heparin (OR 1.78, 95% CI 1.10–2.86, *p* = 0.018) were independently associated with higher complication rates when controlled for other risk factors.

A total of 84 patients (4.1%) developed immediate perioperative complications. Most complications were minor (Table 3). Some immediate perioperative complications included minor oozing from the puncture site (n = 36), small hematoma less than or equal to 5 cm (n = 35), large hematoma greater than 5 cm (n = 4), and puncture site pain (n = 1). Major complications (0.14%) included dissection, which occurred in two patients. In another patient, the access site was lost so a cut-down had to be performed.

**Table 3.** List of complications associated with the use of vascular closure device in our study.

Immediate complications (n = 84)	Number (%)	
*Minor (3.9%)*		
Oozing	36 (42.9)	
Small hematoma (<5 cm)	35 (41.7)	
Large hematoma (>5 cm)	4 (4.8)	
Hematuria	1 (1.2)	
Pain	1 (1.2)	
Emesis	1 (1.2)	
Hives	1 (1.2)	
Bruising	1 (1.2)	
*Major (0.14%)*		
Dissection	2 (2.4)	
Lost access	1 (1.2)	
Not specified	1 (1.2)	
Delayed complications (0.96%)	CT/CTA (n = 3)	Ultrasound (n = 17)
Aneurysm	–	1 (5.9)
Aneurysm + Thrombus	1 (33.3)	1 (5.9)
Leak	–	1 (5.9)
Leak + Thrombus	1 (33.3)	1 (5.9)
Pseudoaneurysm	–	5 (29.4)
Retroperitoneal bleed	1 (33.3)	–
Thrombus	–	2 (11.8)
Small hematoma (<5 cm)	–	2 (11.8)
Large hematoma (>5 cm)	–	4 (23.5)

Three patients had a complication on CT angiogram and 17 patients had a complication on ultrasound performed within 14 days of VCD use. One patient underwent a CT angiogram dissection protocol 11 days after a therapeutic NIR procedure. The study was performed for worsening abdominal pain in the context of a known type B aortic dissection. Although the CT angiogram was performed within the 14-day window, the reason for the imaging was not related to the patient's NIR procedure. One patient had a documented complication, but the progress notes did not specify any further details on the nature of the complication.

## Discussion

VCDs were successfully deployed in 95.2% of the patients with 4.8% of perioperative complications. Most complications were seen in the perioperative period and were minor (3.9%) in nature. VCDs have been shown to significantly improve hemostasis and ambulation after percutaneous vascular procedures, both diagnostic and therapeutic.^1,2,4,5^ Increased patient satisfaction, comfort as well as accelerated ambulation have also been reported.^6,7^ Some studies have shown that certain types of VCDs are 95–100% effective in achieving hemostasis with minimal complications.^3,8–10^ In addition, the use of ultrasound-guidance of femoral artery access has increased the safety and effectiveness of the VCDs.^11^

To our knowledge, our study is the largest single-center real-world experience where the use of VCDs were evaluated for both diagnostic and therapeutic NIR and VIR procedures. This type of practice is unique to our institution and should be considered by other centers for implementation. Despite significant improvements in VCD design, VCDs are not commonly used for all procedures, which may include factors such as cost, but this specific aspect is yet to be determined.^12^ Operator familiarity with the type of VCD likely also plays a role in the increased number of complications and deployment failures. The significantly higher (*p* < 0.001) deployment success rate and the significantly lower (*p* = 0.042) complication rate with therapeutic NIR procedures is likely due to better operator familiarity and more experience of these operators with VCDs. VCDs have been used for therapeutic NIR procedures for far longer when compared to that in other procedures, and there has been significant advancement in procedural techniques over the past 20 years.^13,14^ With more experience in the use of VCDs in VIR procedures and diagnostic NIR procedures, the deployment success will likely become the same. NIR therapeutic procedures are largely comprised of life-altering treatments, such as for stroke patients, which is a global concern.^15^ Particularly, stroke intervention has been revolutionized with early endovascular treatment options available for patients presenting with acute thrombotic or hemorrhagic stroke.^16–18^ Stroke patients are usually brought to the interventional radiology suite with anticoagulation or antiplatelets on board and potentially may be at higher risk of a post-procedural complication if VCDs are used. However, our study does not reach statistical significance in this aspect. One other important point to keep in mind is that delays to stroke treatment during the COVID-19 pandemic have led to decreased mortality and morbidity.^19^ However, patients who receive adequate endovascular treatment and in cases where VCDs are used, can improve patient mobility more quickly, and therefore, be discharged from hospital on a timelier basis also. In principle, a faster discharge would also decrease both hospital and patient costs as well.

There are always potential risks associated with therapeutic NIR procedures, given the higher acuity and nature of patient presentation. Specific neurologic complications may include new or worsening hemorrhage or ischemia, transient changes in level of consciousness, acute intra- or extracranial vessel occlusion/dissection, aneurysm and complications related to equipment.^20^ However, further advances in NIR technique and research has significantly advanced treatment options and also decreased complications.^14^ Our study did not record any neurological complications despite the very high number of procedures performed, which are continuing to increase each year. Operator skill is a contributing factor, but the advances in technique are likely a foundational component to the success.

The Vascular Angio-Seal devices (both 6F and 8F) were the most commonly used VCD in our study and are also one of the most widely used of all VCDs.^21^ Our study showed significantly (*p* < 0.001) higher success in deployment with Angio-Seal compared to the Perclose but no significant (*p* = 0.52) difference in the complications between the two. A study by Patel et al.,^21^ compared multiple different types of VCDs, and found that there was insufficient published evidence or data to favor a specific VCD. A systematic review and meta-analysis by Koreny et al.^22^ showed that the safety and effectiveness of all types of VCDs when compared to manual compression were similar (*p* > 0.13), whether the procedure was diagnostic or therapeutic. However, this particular study focused on coronary angiography/intervention and its complications were specifically related to cardiac catheterization. Hon et al.^23^ also studied the major different types of VCDs, and stated that the choice of device is multifactorial, including availability of the device, operator preference, anticipation of repeat arterial access, and size of the hole, without a significant preference for one type of device over the other. Therefore, the significant result at our institution may simply be related to operator comfort with the Angio-Seal, as it is the most commonly used VCD in our institution.

Our study did not specifically compare the outcome of VCDs with that of manual compression of the puncture site. However, a Cochrane Systematic Review performed in 2016 indicated that suture-based VCDs were associated with reduced time to hemostasis when compared with extrinsic compression and that there was no difference in the incidence of vascular injury or mortality when VCDs were compared with extrinsic compression.^24^ In 2011, a meta-analysis of VCDs in therapeutic VIR procedures showed marginally fewer complications, which was not significantly different (*p* = 0.13), with VCDs compared to manual compression.^25^ A large prospective registry of 13,000 patients, found significantly lower rates of vascular complications with the use of VCDs than with manual compression in “appropriately selected patients undergoing diagnostic and therapeutic cardiac catheterizations”.^26^ Therefore, it can be argued that the benefits from the use of a VCD now outweighs the risks, with a distinct advantage of patient satisfaction and faster time to mobility.^27^ Our study further confirms low rate (4.8%) of predominantly minor complications associated with VCDs. Major complications in our study were seen only in 3 out of 2072 patients (0.14%), where patients had to undergo additional therapeutic procedures for the complications.

Access site hemostasis continues to be challenging for interventional radiologists, particularly in patients who require anticoagulation, antiplatelet, or fibrinolytic drugs. After controlling for other risk factors, patients in our study who required peri-operative use of antiplatelet medications had significantly higher (*p* = 0.024) deployment failure and those with perioperative use of heparin had significantly higher procedural complications (*p* = 0.017). A single-center study performed by Geyik et al.,^28^ found that there were higher rates of major complications in patients who underwent therapeutic NIR procedures who received heparin and/or antiplatelet medication (5.3%). Furthermore, Wong et al.,^29^ revealed that patients treated with VCDs more rapidly achieved hemostasis, with less time for hemostasis, less local hematoma formation, and low level of complications in patients with prolonged ACT after neuro-interventional procedures.

Patients also had significantly higher rates of complications when they had a history of smoking (*p* < 0.001). This is likely secondary to increased vascular risk factors in patients with comorbidities such as smoking, hypertension, diabetes mellitus, and chronic kidney disease, and are innately at higher risk.^8^

Given these findings, it is our hypothesis that the routine use of VCDs in both diagnostic and therapeutic NIR and VIR procedures is generalizable for implementation at other centers. Also, many newer techniques such as using the transradial access approach^30^ and different types of VCDs are being studied in small cohorts.^5^ Currently, our experience with these methods is limited but ongoing vigilant, monitoring of these techniques and devices should be performed.

## Limitations of the study

This study has limitations of a retrospective study. The clinical and procedural details including complications were collected from the documentation by the nursing staff at the time of the patient's procedure. Therefore, this information may have reporting bias. The researchers made every effort to go back and review the clinical and procedural notes, as well as imaging, if any discrepancies were found, but it was difficult to find external data other than the information written in the progress notes. Despite the expected limitations of a retrospective study, our study provides valuable information on a large cohort of patients where VCDs were used for puncture site hemostasis.

## Conclusion

VCDs were successfully deployed in 95.2% of the patients with 4.8% of perioperative minor complications. Hence, VCDs for percutaneous closure of femoral puncture sites are safe and effective for use in both diagnostic and therapeutic interventional radiological procedures.

## References

[1] Noori VJ Eldrup-Jørgensen J . A systematic review of vascular closure devices for femoral artery puncture sites. J Vasc Surg 2018; 68: 887–899. 30146036 10.1016/j.jvs.2018.05.019

[2] Wong SC Bachinsky W Cambier P , et al. A randomized comparison of a novel bioabsorbable vascular closure device versus manual compression in the achievement of hemostasis after percutaneous femoral procedures. The ECLIPSE (Ensure’s Vascular Closure Device Speeds Hemostasis Trial). JACC Cardiovasc Interv 2009; 2: 785–793. 19695549 10.1016/j.jcin.2009.06.006

[3] Aksoy M Becquemin JP Desgranges P , et al. The safety and efficacy of angioseal in therapeutic endovascular interventions. Eur J Vasc Endovasc Surg 2006; 32: 90–93. 16478672 10.1016/j.ejvs.2005.12.014

[4] Heo YJ Jeong HW Baek JW , et al. Ultrasound evaluation of puncture sites after deployment of two different types of vascular closure devices: a prospective comparative study. Cardiovasc Intervent Radiol 2018; 41: 1654–1663. 30120529 10.1007/s00270-018-2056-3

[5] Taha A Walsh EK Wright KA , et al. Safety and feasibility of a novel vascular closure device in neurointerventional procedures. Interv Neuroradiol 2013; 19: 353–358. 24070085 10.1177/159101991301900313 PMC3806011

[6] Brancheau D Sarsam S Assaad M , et al. Accelerated ambulation after vascular access closure device. Ther Adv Cardiovasc Dis 2018; 12: 141–144. 29421959 10.1177/1753944718756604 PMC5941674

[7] Pierot L Herbreteau D Bracard S , et al. An evaluation of immediate sheath removal and use of the angio-seal vascular closure device in neuroradiological interventions. Neuroradiology 2006; 48: 45–49. 16261336 10.1007/s00234-005-0013-8

[8] Chivot C Deramond H Bouzerar R , et al. Safety and efficacy of femoral artery closure with the FemoSeal device after cerebral thrombectomy using an 8 French Sheath. Eur J Vasc Endovasc Surg 2018; 55: 730–734. 29550254 10.1016/j.ejvs.2018.02.014

[9] Kiesz RS Wiernek BK Wiernek SL , et al. Cardiva catalyst II vascular access management device in percutaneous diagnostic and interventional procedures with same-day discharge (CATALYST II Trial). J Endovasc Ther 2011; 18: 46–53. 21314348 10.1583/10-3237.1

[10] Nelson PR Kracjer Z Kansal N , et al. A multicenter, randomized, controlled trial of totally percutaneous access versus open femoral exposure for endovascular aortic aneurysm repair (the PEVAR trial). J Vasc Surg 2014; 59: 1181–1193. 24440678 10.1016/j.jvs.2013.10.101

[11] Sorenson TJ Nicholson PJ Hilditch CA , et al. A lesson from cardiology: the argument for ultrasound-guided femoral artery access in interventional neuroradiology. World Neurosurg 2019; 126: 124–128. 30862603 10.1016/j.wneu.2019.02.171

[12] Applegate RJ Sacrinty MT Kutcher MA , et al. Propensity score analysis of vascular complications after diagnostic cardiac catheterization and percutaneous coronary intervention 1998-2003. Catheter Cardiovasc Interv 2006; 67: 556–562. 16532497 10.1002/ccd.20677

[13] Krishnasamy VP Hagar MJ Scher DJ , et al. Vascular closure devices: technical tips, complications, and management. Tech Vasc Interv Radiol 2015; 18: 100–112. 26070622 10.1053/j.tvir.2015.04.008

[14] Amar AP Lavine SD . Interventional neuroradiology. Neurosurg Clin N Am 2005; 16: 9. 10.1016/j.nec.2005.04.001 15990043

[15] Krishnamurthi R V Feigin VL Forouzanfar MH , et al. Global and regional burden of first-ever ischaemic and haemorrhagic stroke during 1990-2010: findings from the Global Burden of Disease Study 2010. Lancet Glob Heal 2013; 1: e259–e281. 10.1016/S2214-109X(13)70089-5 PMC4181351 25104492

[16] Jansen O Szikora I Causin F , et al. Standards of practice in interventional neuroradiology. Neuroradiology 2017; 59: 541–544. 28526977 10.1007/s00234-017-1837-8

[17] Cox M Atsina KB Sedora-Roman NI , et al. Neurointerventional radiology for the aspiring radiology resident: current state of the field and future directions. Am J Roentgenol 2019; 212: 899–904. 30699013 10.2214/AJR.18.20336

[18] Sacks D van Overhagen H van Zwam WH , et al. The role of interventional radiologists in acute ischemic stroke interventions: a joint position statement from the society of interventional radiology, the cardiovascular and interventional radiology society of Europe, and the interventional radiology society of Australasia. J Vasc Interv Radiol 2019; 30: 131–133. 30385239 10.1016/j.jvir.2018.09.035

[19] Jillella D V Nahab F Nguyen TN , et al. Delays in thrombolysis during COVID-19 are associated with worse neurological outcomes: the Society of Vascular and Interventional Neurology Multicenter Collaboration. J Neurol 2022; 269: 603–608. 34333701 10.1007/s00415-021-10734-z PMC8325534

[20] Davis MC Deveikis JP Harrigan MR . Clinical presentation, imaging, and management of complications due to neurointerventional procedures. Semin Intervent Radiol 2015; 32: 98–107. 26038618 10.1055/s-0035-1549374 PMC4447869

[21] Patel R Muller-Hulsbeck S Morgan R , et al. Vascular closure devices in interventional radiology practice. Cardiovasc Intervent Radiol 2015; 38: 781–793. 25944150 10.1007/s00270-015-1116-1

[22] Koreny M Riedmüller E Nikfardjam M , et al. Arterial puncture closing devices compared with standard manual compression after cardiac catheterization: systematic review and meta-analysis. J Am Med Assoc 2004; 291: 350–357. 10.1001/jama.291.3.350 14734598

[23] Hon LQ Ganeshan A Thomas SM , et al. Vascular closure devices: a comparative overview. Curr Probl Diagn Radiol 2009; 38: 33–43. 19041039 10.1067/j.cpradiol.2008.02.002

[24] Robertson L Andras A Colgan F , et al. Vascular closure devices for femoral arterial puncture site haemostasis. Cochrane Database Syst Rev. 2016; 2016. DOI: 10.1002/14651858.CD009541.pub2 PMC10372718 26948236

[25] Das R Ahmed K Athanasiou T , et al. Arterial closure devices versus manual compression for femoral haemostasis in interventional radiological procedures: a systematic review and meta-analysis. Cardiovasc Intervent Radiol 2011; 34: 723–738. 21046386 10.1007/s00270-010-9981-0

[26] Arora N Matheny ME Sepke C , et al. A propensity analysis of the risk of vascular complications after cardiac catheterization procedures with the use of vascular closure devices. Am Heart J 2007; 153: 606–611. 17383300 10.1016/j.ahj.2006.12.014

[27] Behan MWH Large JK Patel NR , et al. A randomised controlled trial comparing the routine use of an Angio-Seal STS device strategy with conventional femoral haemostasis methods in a district general hospital. Int J Clin Pract 2007; 61: 367–372. 17313602 10.1111/j.1742-1241.2006.01229.x

[28] Geyik S Yavuz K Akgoz A , et al. The safety and efficacy of the Angio-Seal closure device in diagnostic and interventional neuroangiography setting: a single-center experience with 1,443 closures. Neuroradiology 2007; 49: 739–746. 17594084 10.1007/s00234-007-0249-6

[29] Wong HF Lee CW Chen YL , et al. Prospective comparison of Angio-Seal versus manual compression for hemostasis after neurointerventional procedures under systemic heparinization. Am J Neuroradiol 2013; 34: 397–401. 22859279 10.3174/ajnr.A3226 PMC7965118

[30] Pons RB Caamaño IR Chirife OS , et al. Transradial access for diagnostic angiography and interventional neuroradiology procedures: a four-year single-center experience. Interv Neuroradiol 2020; 26: 506–513. 32408785 10.1177/1591019920925711 PMC7446594

